# Stem cell therapy for idiopathic pulmonary fibrosis: a protocol proposal

**DOI:** 10.1186/1479-5876-9-182

**Published:** 2011-10-21

**Authors:** Argyris Tzouvelekis, George Koliakos, Paschalis Ntolios, Irene Baira, Evangelos Bouros, Anastasia Oikonomou, Athanassios Zissimopoulos, George Kolios, Despoina Kakagia, Vassilis Paspaliaris, Ioannis Kotsianidis, Marios Froudarakis, Demosthenes Bouros

**Affiliations:** 1 Department of Pneumonology, Medical School, Democritus University of Thrace, Alexandroupolis, Greece; 2 Department of Biochemistry, Medical School, Aristotle's University of Thessaloniki, Greece; 3 Laboratory of Pharmacology, Medical School, Democritus University of Thrace, Alexandroupolis, Greece; 4 Department of Radiology, Medical School, Democritus University of Thrace, Alexandroupolis, Greece; 5 Department of Nuclear Medicine, Medical School, Democritus University of Thrace, Alexandroupolis, Greece; 6 1st Department of Gebneral Surgery, Medical School, Democritus University of Thrace, Alexandroupolis, Greece; 7 Adistem Ltd., Wanchai, Hong Kong; 8 Department of Hematology, Medical School, Democritus University of Thrace, Alexandroupolis, Greece

**Keywords:** stem cells, adipose tissue, stromal vascular fraction, idiopathic pulmonary fibrosis, therapy, endobronchial infusion, prospective, nonrandomized phase Ib clinical trial

## Abstract

**Background:**

Idiopathic pulmonary fibrosis represents a lethal form of progressive fibrotic lung disorder with gradually increasing incidence worldwide. Despite intense research efforts its pathogenesis is still elusive and controversial reflecting in the current disappointing status regarding its treatment. Patients and Methods: We report the first protocol proposal of a prospective, unicentric, non-randomized, phase Ib clinical trial to study the safety and tolerability of the adipose-derived stem cells (ADSCs) stromal vascular fraction (SVF) as a therapeutic agent in IPF. After careful patient selection based on functional criteria (forced vital capacity-FVC > 50%, diffuse lung capacity for carbon monoxide-DL_CO _> 35% of the predicted values) all eligible subjects will be subjected to lipoaspiration resulting in the isolation of approximately 100- 500 gr of adipose tissue. After preparation, isolation and labelling ADSCs-SVF will be endobronchially infused to both lower lobes of the fibrotic lungs. Procedure will be repeated thrice at monthly intervals. Primary end-point represent safety and tolerability data, while exploratory secondary end-points include assessment of clinical functional and radiological status. Results: Preliminary results recently presented in the form of an abstract seem promising and tantalizing since there were no cases of clinically significant allergic reactions, infections, disease acute exacerbations or ectopic tissue formation. In addition 6 months follow-up data revealed a marginal improvement at 6-minute walking distance and forced vital capacity.

**Conclusions:**

Adipose tissue represents an abundant, safe, ethically uncontested and potentially beneficial source of stem cells for patients with IPF. Larger multicenter phase II and III placebo-controlled clinical trials are sorely needed in order to prove efficacy. However, pilot safety studies are of major importance and represent the first hamper that should be overcome to establish a rigid basis for larger clinical trials.

## Background

IPF is a refractory and lethal form of pulmonary fibrosis characterized by fibroblast proliferation, extracellular matrix deposition, and progressive lung scarring, and comprises the histopathologic pattern of usual interstitial pneumonia (UIP). The incidence of IPF is estimated at 6.8 to 16.3 cases per 100, 000 per year in the United States, and the mean survival from the time of diagnosis is 3 to 5 years regardless of treatment, showing a prognosis worst that than seen in patients lung cancer [1-3].

Although the etiology and pathogenesis of IPF remain poorly understood, current research suggests that the mechanisms driving IPF reflect abnormal, deregulated wound healing in response to multiple sites of ongoing alveolar epithelial injury, involving increased activity and possibly exaggerated responses by a spectrum of proinflammatory and profibrogenic factors [4-10]. So far, despite intense research efforts and clinical trials, there is still no effective treatment that can prolong the survival of patients with IPF [11-14]. Conventional therapeutic approach includes combination of corticosteroids, anti-oxidants, immunosuppressants and immunomodulatory anti-fibrotic agents [11-16]. However, the only, so far, therapeutic approach that has been proven effective in terms of prolonging patient's survival is lung transplantation. Nonetheless, not all the patients with IPF are eligible for lung transplantation whereas there is a significant proportion of these patients that finally succumb while waiting in a lung transplantation list. Therefore, there is critical need for more effective and reliable therapeutic modalities.

Mesenchymal stem cells (MSCs), of different cellular origins (umbilical cord, bone marrow, adipose tissue), represent one of the most challenging and promising areas of novel therapeutic strategies that have been recently applied apart from cosmetic reasons, in several chronic, incurable, fatal diseases such as diabetes mellitus type II, Parkinson's disease, congestive heart failure, renal failure, osteoarthritis and myocardial infarction [17-23].

Of special interest are adipose-derived stem cells (ADSCs)- stromal vascular fraction (SVF), as they present with fruitful therapeutic advantages against bone marrow stem cells including ease of extraction, higher content of MSCs and ex-vivo expandability [21,22]. Currently we have witnessed an explosion of experimental data supporting the migratory, differentiative and reparative capacity of MSCs in animal models of lung inflammation and fibrosis [21,22]. In particular, a number of studies have investigated the role of umbilical cord MSCs as a treatment option in the experimental model of pulmonary fibrosis and showed their potency to be differentiated under a specific micro-environment, into alveolar type II epithelial cells [24] whereas mice adult-stem cells can attenuate lung fibrosis in the bleomycin-model of pulmonary fibrosis [25,26]. Furthermore, repeated intravenous administrations of human and mouse ADSCs resulted in substantial beneficial effects in a model of inflammation and injury induced by cigarette-smoke exposure [27]. More intriguingly a recent report demonstrated for the first time rigid evidence supporting the presence of resident stem cells within human lungs that exert beneficial role in tissue homeostasis and regeneration in animal models [28].

Despite promising therapeutic results arising from experimental studies safety issues and concerns coupled with major controversies regarding IPF pathogenesis, have severely hampered chest physicians' efforts to apply cell-based therapies for the treatment of this dismal disease. To address the above concerns and to establish a rigid basis for future efficacy trials, we are reporting the first protocol proposal of a nonrandomized, unicentric, dose-ranging phase Ib safety study of endobronchial infusion of autologous ADSCs-SVF in IPF patients with moderate disease severity.

### Aim and hypothesis

The aim of the study is to investigate the hypothesis that endobronchial autologous infusion of ADSCs-SVF cells is safe, feasible and well tolerated as a therapeutic modality in IPF patients. Our main hypothesis is that the above procedure can provide to the targeted areas of epithelial injury and excessive fibrotic tissue with adequate number of minimally manipulated multi-potent SVF cells with increased anti-inflammatory paracrine activity as well as regenerative capacity overcoming the fear and concern of undesirable alterations of MSCs during ex-vivo cellular expansion, including contaminations, tumor, ectopic tissue formation and lung toxicity resulting from undesirable engraftment in the microvasculature.

### Patients and methods

In our phase Ib dose escalating study will participate 12 with IPF, respectively, based on the recently published diagnostic criteria of ATS/ERS (2011), of mild to moderate disease severity as estimated functional parameters including forced vital capacity (FVC) > 50% and diffusing lung capacity for carbon monoxide (DL_CO_) > 35% of the predicted normal values. All patients will sign an informed consent form where they will agree to their participation in the study and the anonymous usage of their biological fluids for research purposes (see inclusion criteria below).

### Inclusion criteria

Patients with a histologic or radiologic pattern typical of usual interstitial pneumonia will be included after other causes of fibrosis are ruled out.

The inclusion criteria are:

1. Age 18 to 75 years (both inclusive),

2. A high-resolution computed tomography (HRCT) scan that is very suggestive or consistent with a probable diagnosis of usual interstitial pneumonia.

3. Bronchoalveolar lavage must be performed at any time before inclusion and must have failed to show features supporting alternative diagnoses.

4. The duration of the disease should be more than three months, and bibasilar inspiratory crackles should be present. In addition, the following functional abnormalities have to be present: a dyspnea score of at least 2 on a scale of 0 (minimum) to 10 (maximum), FVC > 50% of the predicted normal value and DLco > 35% of the predicted value.

5. Patients willing to sign an informed consent form where they will agree to their participation in the study and the anonymous usage of their biological fluids for research purposes according to the Declaration of Helsinki.

6. Patients under treatment with n-acetylcysteine or pirfenidone should discontinue drug and enter a wash-out period for at least 6 weeks prior study enrolment.

### Exclusion criteria

1. IPF patients of severe disease based on the baseline functional data, meaning those with FVC < 50% predicted normal value and DLCO < 35%predicted normal value.

2. Patients diagnosed with lung cancer or with an evidence of active malignancy, or prior history of active malignancy that has not been in remission for at least 5 years

3. Patients with documented history of uncontrolled heart, renal or hepatic failure,

4. Patients with serious neurological abnormalities including stroke and myasthenia Gravis

5. Patients under anti-coagulants or in serious emaciation.

6. Patients with active infections.

7. Patients unable to be regularly followed up including those with serious psychiatric disorders.

8. Patients unwilling to sign informed consent form

### Study Methodology

**1) Approval by Local Ethics and Scientific Committee: **Original protocol has already been approved by the Local Ethics and Scientific Committee of the University Hospital of Alexandroupolis, Democritus University of Thrace, Greece (reference number EHD33/1SC/16-02-2010)

**2) Inform consent**: Patients willing to sign an informed consent form where they will agree to their participation in the study and the anonymous usage of their biological fluids for research purposes according to the Declaration of Helsinki.

**Figure 1:**
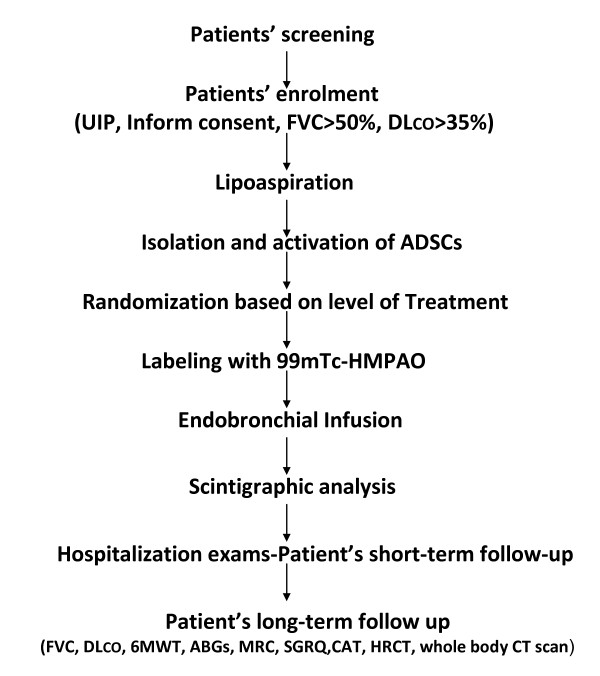
**Step-up procedure of protocol proposal of the endobronchial infusion of ADSCs in patients with IPF starting with patients' screening and enrolment and ending with follow-up period**. Abbreviations: 6MWT: 6-minute walking test, ABGs: Arterial blood gases, ADSCs: Adipose-derived stem cells, CAT: Cough Assessment Test, DL_CO_: Diffuse Lung Capacity for carbon monoxide FVC: Forced Vital Capacity, HRCT: High Resolution Computed Tomography, MRC: Medical Research Council- SGRQ: Saint George's Research Questionnaire, UIP: Usual Interstitial Pneumonia (histologic or radiologic)

**3) Physical and laboratory examination: **All patients will undergo a detailed physical and laboratory examination including: arterial blood gases, electrocardiogram (ECG), estimation of vital signs (blood pressure, temperature, breaths and beats per minute) as well as routine laboratory tests, including white blood cell count and differential count, red blood cell count, liver and renal function, serum C-reactive protein. Patients will undergo spirometry and chest HRCT, at screening to evaluate the functional and radiological severity of the disease and to localize the areas of the lungs where the infusion of SVF cells will be targeted to (Figure 1).

**4) Lipoaspiration and isolation of ADSCs-SVF: **All eligible patients will undergo lipoaspiration under general anesthesia in a sterile surgical operating room setting. 50 mL of blood in EDTA will be taken to prepare platelet rich plasma (PRP). Approximately 100 - 500 gr of adipose tissue will be isolated from the above procedure performed by a plastic surgeon. Enzyme dissolution procedure of the adipose tissue, using collagenase type I solution under agitation for 2 hours plus 10 cc of lecithine followed by two centrifugations at 100 g for 10 minutes the first and at 1800 g for 10 minutes the second, will be performed to separate the stromal cell fraction (pellet) from adipocytes. The pellets will be treated with red lysis buffer. The final pellet will be re-suspended and a small volume of suspension will be used for flow cytometry analysis, counting CD29, CD73, CD90, CD34, CD105, positive cells and CD14, CD31 and CD45 negative cells in Coulter Epics XL/MCL. 10 mL of the suspension will be used for bacterial check with the BacT/Alert system (with colorimetric carbon dioxide detector). In the case of an infected sample the microorganism will be identified with VITEK 2 Compact 15 and will be excluded. A volume of DMSO solution will be added in the remaining suspension so that the final volume contains 10% DMSO and 2% Haes-steril 200. The cells will be cryo-preserved gradually and stored in cryovials, air-tightly sealed with cryoflex membrane. The vials will be placed in constant temperature in liquid nitrogen and will be stored there. Viability of the cells will be estimated by tryphan blue. A total of 3-5 × 10^6 ^cells per gram of adipose tissue is expected to be isolated. Based on the current literature [29], since SVF represents an heterogeneous cell population it is anticipated that approximately 50-70% of the total number of isolated cells to be of mesenchymal origin meaning CD29, CD105, CD90, CD73 positive and CD34, CD45 negative cells and 20-30% mature endothelial (CD31 and positive) and hematopoetic (CD34 positive) cells.

**5) Activation of ADSCs- SVF: **ADSCs- SVF though large in number lie dormant within the adipose tissue and therefore they require activation to come into full functionality for more successful implantation into the host tissue and to begin self-renewal by cell division and formation of other cell types by differentiation and transdifferentiation. In order to increase the anti-inflammatory, angiogenic, anti-apoptotic and perhaps regenerative potential of ADSCs we will apply a two-step activation procedure: a) Activation through autologous PRP. Isolated SVF cells will be subjected to full activation using autologous PRP derived from 50 ml of peripheral blood drawn from each patient. PRP as a storage vehicle of growth factors, is a new application of tissue engineering which was considered for the application of growth factors. PRP is a concentration of platelets in plasma developed by gradient density centrifugation. It contains many growth factors, such as vascular endothelial growth factor (VEGF), epidermal growth factor (EGF), insulin-like growth factor (IGF), Keratinocyte growth factor (KGF), hepatocyte growth factor (HGF) and stromal derived factor (SDF), that may potentially accelerate proliferation of stem cells, improve their chemotactic activity and facilitate their engraftment to the multilayer vessels in order to exert their beneficial properties. b) Activation using low level laser irradiation (5 J/cm^2^). The syringe containing isolated SVF cells enriched with PRP solution will be placed a laser device specifically manufactured for the purposes of this study by Adistem Ltd. Photobiostimulation has been proposed to exert several biological actions including upregulation of angiogenic (VEGF) and anti-inflammatory (IL-1 receptor antagonists, SDF) mediators as well as down-regulation of inflammatory cytokines including tumor necrosis factor(TNF)- alpha, IL-1, 6 and 8 and reactive oxygen species [30] (Figure 1).

**6) Randomization of patients depending on the level of treatment: **To define and standardize the optimal dose regimen that will be both safe and effective we will randomize our 12 patients in the phase I trial into two categories depending on the level of treatment, meaning the number of SVF cells infused per bronchoscopy. In particular we will define as low treatment level the infusion of 0.5 × 10^6 ^SVF cells per kgr of body weight per infusion meaning a total of 1.5 × 10^6 ^SVF cells per kgr of body weight (approximately 120 × 10^6 ^cells in total) and as high treatment level the infusion of 1 × 10^6 ^of SVF cells per kgr of body weight per infusion meaning a total of 3 × 10^6 ^SVF cells per kgr of body weight (approximately 240 × 10^6 ^cells in total). The dose regimen will be based on data arising from previous published studies in patients suffering from myocardial infarction where a dose escalation procedure was applied using a single infusion of 0.5, 1.6 and 5 millions bone marrow mesenchymal stem cells (BM-MSCs) per body weight. Results from the former study demonstrated the absence of a dose-dependent effect while no differences in side-effects between the three studied groups were observed [20]. Moreover, an ongoing clinical trial (the TIME study), with a registration number of NCT00684021, is studying the safety and efficacy of one single dose of intravenously infused 150 million cells in total (meaning 0.5 million cells per kgr of body weight) in patients who had experienced a heart attack. Finally, a recent ongoing clinical trial by Matthay et al [31]. is estimating the safety of two single dose regimens of 5 and 10 million BM -MSCs per kgr of body weight in patients with acute lung injury. Patients in the two randomization groups will be age, sex and disease severity (based on spirometry and HRCT criteria) matched. Our study dose-escalating regimen is in accordance with the existing safety data arising from both published and currently ongoing clinical trials (Figure 1).

**Figure 2:**
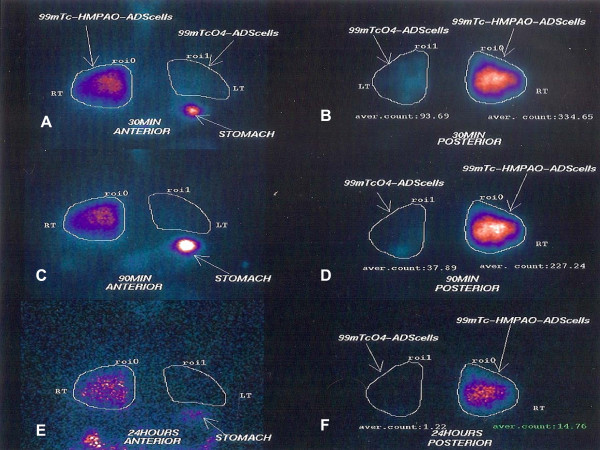
**99mTc lung scintigraphy at different time points (30 min, 90 min and 24 hours) after endobronchial infusion of adipose derived mesenchymal stem cells (ADmSCs)**. Retention of radiolabeled cells (99mTc-HMPAO) (right lung-RT) in comparison to cells with free 99mTcO4 (without ceretec-HMPAO) (left lung-LT) was estimated with computerized image analysis by drawing regions of interest (roi) and calculating the average counts/pixels (aver.count). As depicted in panels A (anterior view) and B (posterior view), signal intensity was significantly stronger in the right lung compared to left lung (roi 334.65 vs. 93.69, respectively), 30 minutes after endobronchial infusion of ADmSCs and this difference remained 90 minutes (panels C and D) (roi 227.24 vs. 37.89, respectively), as well as 24 hours (panels E and F) (roi 14.76 vs. 1.22, respectively) after the infusion, indicating the presence of radiolabeled ADmSCs (99mTc-HMPAO). On the contrary, ADmSCs instilled within the left lung were reconstituted only with free technetium (^99 m^TcO_4_^-^) without ceretec-HMPAO and therefore produced minimal signal intensity within the lung. Free technetium uptake by the stomach is a normal finding due to the presence of free pertechnetate.

**7) Labeling of ADSCs with ^99 m^Tc-HMPAO (^99 m^Tc-ceretec)**: To visualize ADSCs within the lung, in a representative number of patients (n = 4), we will label them with ^99 m^Tc-HMPAO (^99 m^Tc-ceretec) according to a modified protocol [32]. A fraction of isolated ADSCs will be diluted into a 5 cc syringe with sodium chloride 0.9% and incubated with a vial containing 0.5 mg of ceretec reconstituted with approximately 2.0 mCi of free technetium (^99 m^TcO_4_^-^) and prepared to be infused within the right lung. A second syringe containing unlabeled ADSCs and 5 mL sodium chloride 0.9% reconstituted with approximately 2.0mCi ^99 m^TcO_4_^- ^and prepared to be administered into the left lung serving as control sample. As depicted in Figure 2 and as was expected, signal intensity was much stronger in the right lung and this effect remained even 24 hours after the infusion, indicating the presence of ADSCs (Figure 1).

**8) Endobronchial Infusion: **The patient will then undergo bronchoscopy using a flexible bronchoscope, under local anesthesia (xylocaine). The flexible bronchoscope will be guided into the lower lobes of both lungs and 1 aliquot of 10 cc SVF cells will be infused using a small catheter (2.0 mm of diameter) through the bronchoscopic channel. Procedure will be repeated thrice at monthly intervals (Figure 1).

**9) Scintigraphic analysis: **Planar imaging of the lungs in anterior and posterior position will be performed 30 min, 90 min and 24 hours after the administration. At 24 hours single photon emission tomography (SPECT) imaging of the lungs will be also done. Imaging will be implemented in a single-head gamma-camera (GE Millennium MPS, Milwaukee USA) equipped with a low energy high resolution (LEHR) parallel-hole collimator. For planar imaging the matrix size will be set at 256 × 256, while for SPECT at 128 × 128 pixels; the photopeak will be centered at 140 keV, with a symmetrical 20% window. The tomographic imaging parameters consisted of a 360°-rotation angle, a circular technique and an acquisition time of 30 sec per frame. Raw imaging data will be reconstructed using the Butterworth-filtered back-projection algorithm, generating tomographic views of the lungs in the 3 planes (transverse, coronal, and sagittal). In order to estimate the retention of the radiolabeled cells in the lung in comparison to free technetium we will draw regions of interest (ROI's) over the right and left lung in the posterior images at 30, 90 min and 24 hours (Figure 2).

**10) Patient's discharge: **The patient will be discharged 24 hours post-bronchoscopy given that he/she is afebrile and hemodynamically stable, with no signs of infection or any type of allergic reaction.

**11) Clinical, functional and radiological assessment: **Arterial blood gases (ABGs) coupled with clinical (Borg and Medical Research Council-MRC dyspnea scales), health-related quality of life (Saint George's Research Questionnaire-SGRQ and cough assessment test-CAT) and functional (6-minute walking test-MWT, FVC and DL_CO_) assessment will be performed every month after the first infusion until the end of the follow-up period (12 months) and HRCT evaluation 6 months after the first infusion in order to estimate any potential clinical, functional and radiological differences compared to baseline modalities (Table 1).

**Table 1:** Scheduled visits following endobronchial infusion of ADScs

No of Visit	Time-point (days)	ADSCs infusion	Physical Examination	Routine Laboratory tests	MRC dyspnea scale	SGRQ + CAT	6MWT	PFTs	ABGs	HRCT
1	0	1^st^	X	X	X	X	X	X	X	X
2	30	2^nd^	X	X	X	X	X	X	X	
3	60	3^rd^	X	X	X	X	X	X	X	
4	90		X	X	X	X	X	X	X	
5	180		X	X	X	X	X	X	X	X
6	270		X	X	X	X	X	X	X	
7	360		X	X	X	X	X	X	X	X + whole body HRCT scan

**Abbreviations: **6MWT: 6-minute walking test, ABGs: Arterial blood gases, ADSCs: Adipose-derived stem cells, CAT: Cough Assessment Test, HRCT: High Resolution Computed Tomography, PFTs: Pulmonary Function Tests, SGRQ: Saint George's Research Questionnaire.

### Primary end-points (Safety and Toxicity levels)

The procedure is safe and well tolerated by the patients, as reflected by the absence of signs compatible with infection, allergic reaction, disease acute exacerbation, and ectopic tissue formation, at 12 months after the first post-endobronchial infusion of SVF cells (total follow-up duration for each patients = 12 months).

In particular, patients will be subdivided into three categories depending on the level of toxicity, defined as:

**1) Low level**, including minor side effects such as increased cough, low fever (less than 37.5°C, skin allergic reactions)

**2) Medium level**, including non-life threatening allergic reactions, infections that do not require hospitalization, elevation of liver enzymes or creatinine serum levels.

**3) High level**, including death and/or life threatening major adverse events such as disease acute exacerbations defined as follows:

*a) *If the patient experiences a significant worsening of dyspnea and a decline in exercise tolerance associated with a 10% decrement of their FVC, a 10 mmHg change in PaO_2 _or 50% increase in their oxygen requirement, and a significant progression in their radiographic score between the three month clinic visits;

*b) *If the patient needs to be admitted to the hospital because of worsening respiratory failure requiring mechanical ventilation, worsening gas exchange, or presumed pulmonary infection.

*c) *If the patient develops ectopic tissue formation compatible with carcinogenesis as assessed by whole body computed tomography at 12 months after the first post-endobronchial infusion of SVF cells

Exploratory secondary end-points:

1) Assessment of Forced Vital Capacity (FVC%) predicted.

2) Assessment of Diffuse Lung Capacity for carbon monoxide (DL_CO_%) predicted.

3) Assessment of 6MWT.

4) Assessment of dyspnea scales (Borg, MRC) as well as health-related quality of life questionnaires (SGRQ)

5) Assessment of the disease extent and severity as reflected by chest high resolution computed tomography (HRCT) at 6 and 12 months post-bronchoscopy.

*HRCT (scoring grade 0 = no lesion, grade 1 = less than 5% of the lobe, grade 2: 6-25% of the lobe, grade 3: 26-50% of the lobe, grade 4 = 51-75% of the lobe, grade 5 = 76-100% of the lobe). The "ground-glass" alveolitis will be defined as increased opacification of the lung without obscuration of the bronchial and vascular markings, whereas the fibrosis score will include thickening of the alveolar septa and subpleural areas of honeycombing.

### Statistical analysis

Statistical analysis will be performed using the SPSS 16.0 software. Safety and exploratory efficacy secondary end-points will be observed for each patient against the baseline values. A p value < 0.05 will be considered as statistically significant.

### Basic Principles & Ethical Considerations

The study will be performed in accordance with Institutional Review Board and the Ethics and Scientific Committee of the University Hospital of Alexandroupolis, Democritus University of Thrace standards. Original protocol has already been approved by the Local Ethics and Scientific Committee of the University Hospital of Alexandroupolis, Democritus University of Thrace, Greece (reference number EHD33/1SC/16-02-2010)

Study Duration & Termination

The clinical part of the study will start after the approval of the responsible Ethics Committee and the study initiation visit is performed.

The time frame of the study will be as follows:

• Total Clinical Duration: 24 months

• Recruitment Period of Phase Ib (12 subjects): 12 months

• Treatment + follow-up (per subject): 12 months

The entire study may be prematurely terminated/discontinued if there is:

1. Comprehensive deficiency in the recorded data or protocol compliance so that the study cannot be reliably assessed

2. Occurrence of undesired experiences or adverse events necessitating termination (high toxicity level).

3. The Principal Investigator and the Project Manager commonly decide premature termination, after consultation with the Ethics Committee.

In the event of early termination, Investigator will cease use of the investigational drug immediately. A written statement fully documenting the reasons for such a termination will be provided from the Department of Pneumonology, Democritus University of Thrace to the Institutional Review Boards/Independent Ethics and Scientific Commitees and Regulatory Authorities of the University Hospital of Alexandroupolis, Democritus University of Thrace, Greece.

### Completed

The subject completes the study as per protocol at 12 months follow up after the first infusion of SVF cells and a whole-body CT scan will be performed at the end of the follow-up period to estimate the presence or not of ectopic tissue formation.

### Discontinued/withdrawn

Withdrawal from the study can occur under the following circumstances:

1. Death

2. Intolerable toxicity during infusion of test drug

3. Subject consent withdrawal

4. Investigator's discretion

5. Screening errors: From an analysis perspective, a 'screening error' is any subject who was enrolled into the study, e.g. was attributed a CRF with subject number, but was withdrawn (before or after randomization) prior to administration of IP for reasons such as protocol violation (inclusion/exclusion criteria) or withdrawal of consent.

### Recording Subject Withdrawals

In the event of a subject withdrawal, the following information must be recorded in the Case Report Form (CRF):

1. Date of withdrawal

2. Reason for terminating participation in the study

3. Last treatment date

4. Follow-up assessments

The Investigator should continue to follow-up all serious adverse events or other adverse events that are considered to be related to the study drug, until they are resolved or assessed to be chronic or stable by the Investigator. This should be documented in the subject's medical records. This follow-up may be extended beyond the end of the study period.

### Replacement of Subjects Withdrawn

Subjects who are prematurely withdrawn from the study will not be replaced.

## Discussion

Currently, the application status of MSCs as treatment modalities in IPF is still in its infancy and remains exploratory. Although a number of safety and efficacy clinical trials of MSCs as therapeutic options in immune-mediated and cardiac diseases have already been published with tantalizing results, to our disappointment, pulmonary and critical care medicine have traditionally lagged behind other therapeutic and research fields including hematology, gastroenterology and cardiology in translational studies of the use of reparative cells [21-23,33,34]. Standing in front the dilemma something or nothing the agony and at the same time desire of chest physicians to test novel therapeutic options for patients with IPF is clearly understandable. MSCs and especially ADSCs may provide an ethically uncontested, cost-effective, easily accessible and well tolerated therapeutic option in the hands of chest physicians in the following years. For these cell-based therapies to become truly evolutionary there is only one approach: phase I safety clinical trials followed by large multicenter randomized controlled efficacy clinical trials [21].

Nevertheless safety concerns mainly arising from IPF pathogenesis that is still elusive and controversial coupled with issues reflecting the origin and the minimal potential of ADSCs to differentiate into fibroblastic colony forming units have severely hampered clinicians' efforts to apply, so far, cell-based therapies for the treatment of this fatal disease [35,36].

To address the above concerns and to establish a rigid basis for future efficacy clinical trials we propose for the first time in the therapeutic field of IPF a protocol referring to a phase Ib nonrandomized clinical trial to evaluate safety and feasibility of endobronchial infusion of autologous ADSCs-SVF in patients with IPF of mild to moderate disease severity. Original protocol has already been approved by the Local Ethics and Scientific Committee of the University Hospital of Alexandroupolis, Democritus University of Thrace, Greece (reference number EHD33/1SC/16-02-2010) with the title "A prospective, unicentric, non randomized, phase I clinical trial to study the safety of the adipose-derived adult stromal vascular fraction cells as a therapeutic agent in Idiopathic Pulmonary Fibrosis".

So far preliminary data recently presented at the 21^ST ^European Respiratory Society Congress, Amsterdam 2011 in the form of an abstract seem promising and intriguing since none of the 12 patients enrolled in the study experienced any clinically significant cases of infections, allergic reactions, acute exacerbations or other major side effects requiring hospitalization. In addition 6 months follow-up data revealed a marginal improvement at 6MWD and FVC levels [21,37].

The main purpose of this project and consequently this trial is to provide worldwide scientific committee with pivotal safety data regarding this novel therapeutic option and accelerate its application to a larger cohort of patients in the context of multicenter phase II and III clinical trials. There is plenty of room for further technical improvements, development and widespread acceptance and accessibility. However, pilot safety studies are of major importance and represent the first hamper that should be overcome to establish a rigid basis for larger clinical trials where safety concerns will be addressed cautiously and thoroughly and efficacy outcomes of high scientific rigidity will be safely extracted.

## Abbreviations list

6MWT: 6-minute walking test; ABGs: Arterial blood gases; ADSCs: Adipose-derived stem cells; BM-MSCs: Bone Marrow-Mesenchymal Stem Cells; CAT: Cough Assessment Test; CRF: Case Report Form; DL_CO_: Diffusing lung capacity for carbon monoxide; ECG: Electrocardiogram; FVC: Forced Vital Capacity; HGF: Hepatocyte Growth Factor; HRCT: High Resolution Computed Tomography; IGF: Insulin-like Growth Factor; KGF: Keartinocyte Growth Factor; IPF: Idiopathic Pulmonary Fibrosis; PFTs: Pulmonary Function Tests; PRP: Platelet Rich Protein; SDF: Stromal derived Factor; SGRQ: Saint George's Research Questionnaire; SVF: Stromal Vascular Fraction; UIP: Usual Interstitial Pneumonia, VEGF: Vascular Endothelial Growth Factor.

## Competing interests

The authors declare that they have no competing interests.

## Authors' contributions

AT and DB designed the protocol and participated in writing the manuscript. GK, PN, IB, EB, AO, AZ, NB, GK, VP, DK, IK and MF contributed in writing, editing and reviewing the manuscript for important intellectual content. All authors read and approved the final version of the manuscript.
